# Development of an Fe^2+^ sensing system based on the inner filter effect between upconverting nanoparticles and ferrozine†

**DOI:** 10.1039/d3ra04645a

**Published:** 2023-09-04

**Authors:** Ruth Abramson, Hannah Wilson, Marta M. Natile, Louise S. Natrajan

**Affiliations:** a Department of Chemistry, School of Natural Sciences, The University of Manchester Oxford Road Manchester M13 9PL UK louise.natrajan@manchester.ac.uk; b Institute of Condensed Matter Chemistry and Technologies for Energy (ICMATE), National Research Council (CNR) c/o Department of Chemical Sciences, University of Padova Via F. Marzolo 1 35131 Padova Italy; c Photon Science Institute, The University of Manchester Oxford Road Manchester M13 9PL UK

## Abstract

The ferrozine (FZ) assay is a vital oxidation state-specific colorimetric assay for the quantification of Fe^2+^ ions in environmental samples due to its sharp increase in absorbance at 562 nm upon addition of Fe^2+^. However, it has yet to be applied to corresponding fluoresence assays which typically offer higher sensitivites and lower detection limits. In this article we present for the first time its pairing with upconverting luminescent nanomaterials to enable detection of Fe^2+^*via* the inner filter effect using a low-power continuous wave diode laser (45 mW). Upon near infra-red excitation at 980 nm, the overlap of the upconversion emission of Er^3+^ at approximately 545 nm and the absorbance of the FZ:Fe^2+^ complex at 562 nm enabled measurement in the change of UCNP emission response as a function of Fe^2+^ concentration in a ratiometric manner. We first applied large, ultra-bright poly(acrylic acid) (PAA)-capped Gd_2_O_2_S:Yb^3+^,Er^3+^ UCNPs upconverting nanoparticles (UCNPs) for the detection of Fe^2+^ using FZ as the acceptor. The probe displayed good selectivity and sensitivity for Fe^2+^, with a low limit of detection (LoD) of 2.74 μM. Analogous results employing smaller (31 nm) PAA-capped hexagonal-phase NaYF_4_:Yb^3+^,Er^3+^ UCNPs synthesised in our lab were achieved, with a lower LoD towards Fe^2+^ of 1.43 μM. These results illustrate how the ratiometric nature of the system means it is applicable over a range of particle sizes, brightnesses and nanoparticle host matrices. Preliminary investigations also found the probes capable of detecting micromolar concentrations of Fe^2+^ in turbid solutions.

## Abstract 2

Addition of Fe^2+^ to a solution of ferrozine and Er^3+^-doped upconverting nanoparticles induces a clear yellow to purple colour change, and excitation at 980 nm causes a decrease in the intensity of the green upconversion visible emission band.

## Graphical abstract

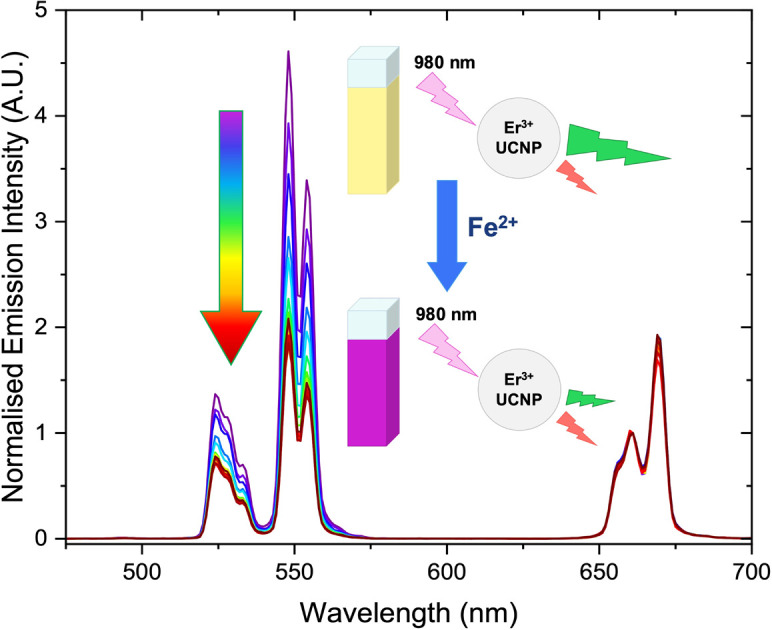

## 1. Introduction

Iron is an essential nutrient that plays many vital roles in the human body in both its ferrous (Fe^2+^) and ferric (Fe^3+^) oxidation states.^1^ Iron in the body is typically found bound in metalloproteins, but a small amount exists as non-chelated iron ions within a ‘labile iron pool’.^2^ Imbalances in this pool have been linked to neurodegenerative diseases and cancers, largely due to the ability of iron to generate the ˙OH radical *via* the Fenton reaction, thought to be the most dangerous reactive oxygen species (ROS) found in cells.^3^ It is therefore of paramount importance that we are able to detect and accurately quantify the amount of iron present in biological samples to prevent toxicity.

Ferrozine (FZ) is a well-known colorimetric assay for Fe^2+^ which was first synthesised and applied to the detection of ferrous iron in 1970.^4^ Aqueous solutions of FZ are pale yellow in colour and display weak absorbance in the visible region. However, upon addition of Fe^2+^ ions, a deep purple 3 : 1 FZ:Fe^2+^ complex is formed with a molar extinction coefficient of 27 900 M^−1^ cm^−1^ at 562 nm. In the literature, ferrozine is frequently applied to accurately detect nanomolar concentrations of Fe^2+^ in environmental settings such as seawater and soils, and has the advantages of being operable at ambient temperatures and pH values close to neutral.^4,5^

In recent years there has been an increased interest in the development of metal ion probes based on emission response as opposed to absorbance, due to powerful yet portable laser systems becoming more cheaply and readily available. Fluorescent probes are also applicable in biological samples, including live cells, without the need for further preparation steps to form a homogenous solution necessitated by the use of colorimetric probes.^3^ Pairing colorimetric or fluorescent dyes with emissive donors allows detection of analytes *in situ*, with minimal further sample preparation steps.^6^ While several ‘turn-on’ fluorescent probes for Fe^2+^ have been reported, such as RhoNox-1 and Rh-T, in our hands, they are typically difficult to synthesise and expensive to purchase even in small amounts.^2,7^ On the contrary, FZ is relatively cheap to purchase in bulk from any chemical supplier, allowing for development of a more readily accessible fluorescent probe for Fe^2+^. Furthermore, the sharp increase in absorbance upon addition of an analyte and its high molar absorption coefficient relative to other colorimetric Fe^2+^ probes means that FZ is well-suited to acting as the acceptor in a luminescent sensing system.^8^ Luminescent nanomaterials, such as quantum dots and inorganic nanoparticles, are of particular interest for use as emissive donors, thanks to their narrow emission bands compared to organic fluorophores and large Stokes/anti-Stokes shifts.^9,10^

Lanthanide-doped upconverting nanoparticles (UCNPs) are an attractive platform for luminescence sensing of analytes. First synthesised in 2004, they have since been widely applied as luminescent phosphors in a multitude of settings including bioimaging, photodynamic therapy (PDT) and drug delivery.^11–16^ Typically, UCNPs employ Yb^3+^ as the sensitiser ion, which is excited by near infra-red (nIR) light at 980 nm (^2^F_5/2_ ← ^2^F_7/2_).^17^ This transition is resonant with intra f–f transitions in Er^3+^, Tm^3+^ and Ho^3+^, which are co-doped into the nanoparticle host matrix as activator ions alongside the Yb^3+^ ions. Subsequent energy transfer from Yb^3+^ leads to sequential, multi-step excitation of the activator ions, resulting in emission in the visible region *via* energy transfer upconversion, made possible by the long-lived excited states of Ln^3+^ ions.^18,19^ Excitation in the nIR is particularly advantageous, particularly where samples are sensitive to shorter wavelengths such as biological samples, due to its greater penetration depth through biological tissue and turbid solutions relative to visible and UV light, lack of biological autofluorescence, and nIR light is less photo-damaging than higher energy wavelengths of light.^20,21^ Furthermore, the resultant emission bands are sharp and narrow with a high signal-to-noise ratio due to the discrete, ladder-like spin–orbit coupled electronic excited states of the Ln^3+^ dopant ions.^22^ The host matrices of UCNPs are typically fluoride or oxide based, both of which offer high chemical stability and low bio- and cytotoxicity.^23,24^

The overlap of the UCNP emission bands with the absorbance of various fluorescent molecular probes has led to the increased popularity of UCNPs in the field of sensing, mostly *via* resonance energy transfer (RET) phenomena.^25,26^ In this process, the donor and acceptor species must have a significant spectral overlap of their emission and absorption, respectively, so that upon excitation of the donor species, this energy can be transferred to the acceptor. Upon addition of an analyte of interest, the absorbance of the acceptor species changes in such a way that the spectral overlap with the donor emission changes, and this change can be detected spectroscopically.^6,27^ Since RET processes rely on energy transfer, they are therefore distance dependent, which in the case of UCNPs typically means that covalent attachment of the acceptor to the UCNP surface is required.^28,29^

Inner filter effects (IFE), which refer to the absorbance of emitted light by an acceptor, are often considered to be a source of error in fluorescence measurements, but with UCNP-based probes it presents an attractive alternative mechanism applicable for sensing applications, as a change in emission intensity of the donor is still able to be detected *via* photoluminescence spectroscopy.^30^ Due to its simplicity and ease of implication, several UCNP-based sensing systems utilising IFE have been published in the literature for important analytes including NO_2_^−^, Ag^+^, Sn^2+^, Cr^6+^, F^−^, S^2−^ and various different biomolecules.^31–38^

Here, we report a new upconversion system for the detection of Fe^2+^, based on IFE between two different Er^3+^-doped upconverting nanophosphors and FZ using a low-power laser as the excitation source. The system is highly selective and sensitive towards Fe^2+^ over other common competing ions. To the best of our knowledge, this is the first time FZ has been used in conjunction with luminescent nanomaterials and presents promising first steps towards a fluorescent sensor based on the readily available reagent FZ using low energy nIR excitation wavelengths. We also suggest some potential future work that could improve the overall sensitivity of the probes, particularly in biological samples.

## 2. Materials and methods

### 2.1 Materials

Gd_2_O_2_S:Yb^3+^,Er^3+^ UCNPs (PTIR545) were kindly donated to us by Phosphor Technology Ltd. (UK). For the synthesis of NaYF_4_:Yb^3+^,Er^3+^ UCNPs, Y_2_O_3_, Yb_2_O_3_, Er_2_O_3_ and NaOH were all purchased from Sigma Aldrich UK. Oleic acid, 1-octadecene and NH_4_F were acquired from Fisher Scientific UK. For the post-synthesis poly(acrylic acid) (PAA)-capping of the UCNPs, both NOBF_4_ and PAA were purchased from Sigma Aldrich UK. Ferrozine sulphate hydrate, Fe^2+^ sulphate and MES were purchased from Sigma Aldrich UK, and all salts of competing metal ions were obtained from either Sigma Aldrich UK or Fisher Scientific UK. For the turbid solution measurements, milk powder was purchased from Sainsbury's (UK). The TEM grids used for characterisation were purchased from Agar Scientific (UK).

### 2.2 Synthesis

#### 2.2.1 Hexagonal-phase NaYF_4_:Yb^3+^,Er^3+^ UCNPs

Rare-earth (RE) oxides (0.39 mmol Y_2_O_3_, 0.1 mmol Yb_2_O_3_, 0.01 mmol Er_2_O_3_) were heated to reflux temperature in dilute hydrochloric acid for 6 hours, and the excess acid evaporated to afford the corresponding RE chlorides. Oleic acid (6 mL) and 1-octadecene (15 mL) were added and the solution was heated to 150 °C under a weak vacuum for 30 minutes. The solution was then cooled to room temperature, at which point a second monomer solution containing NaOH (2.5 mmol) and NH_4_F (4 mmol) dissolved in 10 mL anhydrous MeOH was quickly injected into the reaction flask. The solution was left to stir under argon for 30 minutes at room temperature before heating to 300 °C (heating rate approx. 10 °C min^−1^) for 65 minutes, during which the temperature was carefully controlled. The reaction was then cooled to room temperature, and the resultant nanoparticles precipitated with EtOH before washing ×3 in EtOH and hexane (4 : 1 ratio, 8 mL total) *via* centrifugation at 4500 rpm. The final product particles were stored at 4 °C under EtOH.

#### 2.2.2 PAA-capping of UCNPs

UCNPs (10 mg) were dispersed in cyclohexane (1.5 mL) and a solution of NOBF_4_ in DMF (10 mg mL^−1^, 1.5 mL) was added. The solution was stirred vigorously, then left to settle. The top cyclohexane layer was removed and the DMF layer washed 3 times with chloroform (4 : 1 ratio) by centrifugation at 4500 rpm. The UCNPs were then dispersed in deionised water (1 mL) and a solution of PAA (20 mg mL^−1^, 1 mL) (*M*_w_ = 1800 g mol^−1^) was added. The solution was stirred for 1.5 hours, then washed twice with EtOH and once with deionised H_2_O *via* centrifugation. The resulting PAA-capped UCNPs were stored under deionised H_2_O at 4 °C.

### 2.3 Characterisation

#### 2.3.1 Transmission electron microscopy (TEM)

Nanoparticle samples were prepared *via* suspension in toluene (1 mg mL^−1^) before drop-casting onto 3 mm carbon-film coated copper mesh grids and air-drying. Images were obtained using an FEI Tecnai G2 F20 TEM equipped with a CCD camera and were processed using the Gatan DigitalMicrograph and ImageJ software.

#### 2.3.2 Powder X-ray diffraction (pXRD)

Samples were prepared by air-drying and grinding into a fine powder using an agate pestle and mortar. Diffraction patterns were obtained using a Phillips PANalytical X'Pert diffractometer. Samples were measured between 10–90° with a scanning speed of 0.047° s^−1^. Data was analysed with the OriginPro® software.

### 2.4 Detection of Fe^2+^ with FZ

Stock solutions of FZ sulphate hydrate and Fe^2+^ sulphate were prepared fresh in deionised water on the day of measurements. Buffer (MES, 0.1 M, pH 5.6) was prepared fresh as required. Nanoparticle dispersions were sonicated for 10 minutes prior to measurement to break up any aggregation. All experiments were conducted in quartz cuvettes (path length 1 cm) using a Mettler Toledo UV5Bio UV-vis spectrometer (scan time 10 seconds) and an Edinburgh Instruments FLSP920 phosphorimeter with a custom-built 980 nm CW diode laser (45 mW). Excitation bandwidth was non-variable, and the emission bandwidth was set to 4 nm (unless stated). Spectra were recorded with a dwell time of 0.1 s, a step size of 1 nm and 3 repeats, with excitation correction files applied.

For colorimetric UV-vis absorbance experiments, FZ (100 μM) was added to MES buffer and aliquots of metal stock solution were added and absorbance measured after each addition. Care was taken to not add more than 10% of the volume in the cuvette to avoid significant dilution effects. For upconversion emission studies, PAA-capped Er^3+^-doped UCNPs were also added to the cuvette (150 μL of a 10 mg mL^−1^ suspension in deionised water). As with the colorimetric sensing studies, aliquots of Fe^2+^ sulphate stock solution were added, and emission measured after each addition. All data were analysed using both Microsoft Excel and OriginPro®.

### 2.5 Competing ion titrations

Solutions of competing metal ion salts, as well the as FZ stock solution, were prepared fresh on the day of measurement. Measurements were conducted using the same method as above, but with salts of each competing ion added as opposed to Fe^2+^ sulphate. Data were analysed using Microsoft Excel and OriginPro®.

### 2.6 Detection of Fe^2+^ in turbid solutions

Stock solutions of Fe^2+^ sulphate and FZ were prepared as previously described, and a suspension of milk powder in water (0.1 g mL^−1^) was prepared. Measurements were conducted using a Spectra-Physics Mai Tai® HP Ti:sapphire ultra-fast fs pulsed laser (average power at 980 nm = 1.35 W), with a neutral-density filter (2.0) used to attenuate average power to approximately 19 mW. Measurements were conducted using deionised water as the solvent to prevent dissolution of milk (note that FZ is still efficient at neutral pH's).^4^ Milk powder suspension (50 μL) was added to the cuvette alongside the FZ and PAA-capped UCNPs to form a cloudy, turbid solution prior to measurement, and Fe^2+^ stock solution stock solution was added as previously described. The pH was measured before and after each experiment to confirm that there was no change. Data were collected using the SpectraSuite software (10 scans, with an integration time of 400 ms) and analysed using Microsoft Excel and OriginPro®.

## 3. Results and discussion

### 3.1 Synthesis of NaYF_4_:Yb^3+^,Er^3+^ UCNPs

Two different categories of UCNPs were applied in this study. The first of the iron quantification titrations were conducted using Gd_2_O_2_S:Yb^3+^,Er^3+^ (referred to as PTIR545 throughout) nanoparticles, kindly donated to us by Phosphor Technology Ltd. Analysis has shown these to have a both a much larger average diameter and wider size distribution than UCNPs typically synthesised in our lab, but this comes with the advantage of superior brightness, resulting in more well-resolved spectra when measured with the 980 nm CW diode laser.

**Fig. 1:**
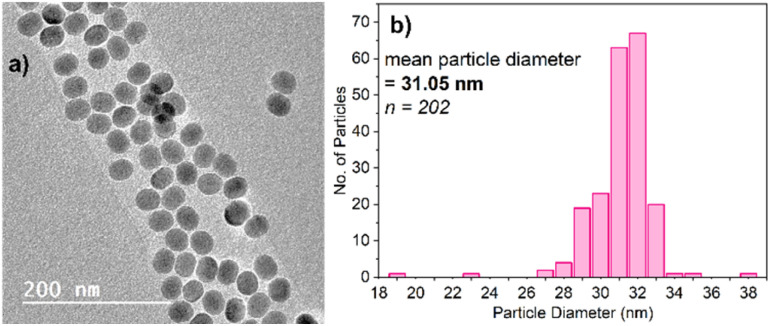
(a) TEM image of the NaYF_4_:Yb^3+^,Er^3+^ UCNPs as synthesised. Note the brightness and contrast of the image have been slightly edited for clarity, but no further changes have been made. (b) The size distribution of the nanoparticles based on diameter derived from particle area.

Titrations were also conducted using PAA-capped NaYF_4_:Yb^3+^,Er^3+^ UCNPs synthesised as described herein, since these are arguably the UCNPs most prevalent in the literature. This allows for comparison between the commercial PTIR545 nanoparticles and those that are more readily accessible to any laboratory with the materials and expertise available for nanoparticle synthesis. The synthesis of NaYF_4_:Yb^3+^,Er^3+^ UCNPs was conducted using an adapted version of the ‘user-friendly’ synthesis pioneered by Li and Zhang in 2008, due to its simplicity and lack of toxic by-products.^39^Fig. 1 shows the characterisation of these UCNPs by TEM, as well as their size distribution. The TEM images show their morphology is largely spherical, allowing average diameter to be derived from particle area. Average particle diameter was calculated as approximately 31 nm, with a relatively high degree of monodispersity throughout the sample. Size and morphology control is achieved by careful control over the reagent ratios and temperature throughout the synthesis.^40,41^ Powder X-ray diffraction showed that the NaYF_4_ host matrix is hexagonal in phase, which has been found to give the most efficient upconversion luminescence compared to the cubic phase (Fig. S3†).^11^ This is achieved by heating at elevated temperatures for extended time periods to achieve full conversion from the cubic phase to the hexagonal phase.^42,43^

The UCNPs as synthesised are capped with oleic acid, which aids in stabilising the nanoparticles in non-polar solvents and preventing aggregation, but utilisation of the UCNPs in aqueous environments such as buffers requires post-synthesis ligand substitution. Poly(acrylic acid) was chosen due to its relatively low cost, its lack of interaction with FZ, and the abundance of reliable methods for ligand substitution available in the literature.^44–46^ The modified particles were easily dispersible in water, with minimal aggregation or settling over time. Analysis by FTIR spectroscopy further showed successful substitution of the capping ligand, as demonstrated by the reduced intensity of the sharp C–H stretches between 2800–3000 cm^−1^ (Fig. S6†), although recording spectra of suspended nanoparticles (as opposed to solid dried-out particles) has resulted in some loss of detail. The major disadvantage of post-synthesis ligand substitution is a loss of luminescence intensity, largely due to an increased deactivation of the luminescence by surface ligand and solvent vibrations, as well as the attenuation of excitation light due to the absorption of water at 980 nm.^47^ However, the luminescence intensity of the PAA-capped NaYF_4_:Yb^3+^,Er^3+^ UCNPs was still found to be sufficient for the iron detection experiments upon excitation with a low-power 980 nm diode laser (45 mW), and the ratiometric nature of the probe enabled comparison with the PAA-capped commercial PTIR545 UCNPs.

### 3.2 Absorbance response

Initially, we conducted titrations demonstrating the absorbance response of FZ to Fe^2+^. Fresh solutions of both FZ and Fe^2+^ sulphate were prepared on the day of the experiment, with all repeats conducted on the same day. Due to this, no extra reducing agents commonly seen in the literature surrounding the FZ assay (such as ascorbic acid) were added since the rate of auto oxidation of Fe^2+^ to Fe^3+^ is sufficiently slow that it did not significantly affect the results.^5,48–50^ Furthermore, solutions were not degassed so as to more closely resemble intended analysis in the presence of air; previous studies have not found the presence of O_2_ to significantly affect the response.^49^

**Fig. 2:**
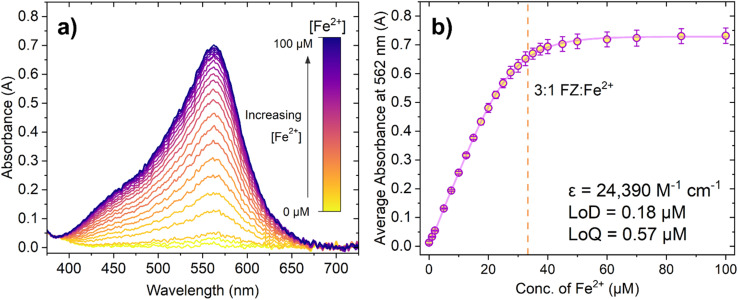
(a) Absorbance spectra of ferrozine (100 μM) in pH 5.6 MES buffer (0.1 M) with increasing concentration of Fe^2+^ ions. Note that this spectrum shows an example of one repeat, and three repeats were taken overall. (b) The average absorbance at the maximum (562 nm) taken over three repeat measurements, with error bars showing standard deviation. The dashed orange line indicates the concentration of Fe^2+^ at which the 3 : 1 FZ:Fe^2+^ complex is expected to form (approx. 33.33 μM Fe^2+^). Data fitted using a biphasic dose response function in OriginPro®.

Fig. 2 shows how increasing the concentration of Fe^2+^ causes a dramatic increase in absorbance in the peak centred at 562 nm, up until a certain point where there is minimal further change. This is observed more clearly in Fig. 2b, where the response begins to plateau after approximately 45 μM Fe^2+^ has been added. The dashed vertical line at 33.33 μM Fe^2+^ represents the concentration of Fe^2+^ at which the 3 : 1 FZ:Fe^2+^ complex should have, theoretically, fully formed and the solution should be fully saturated with purple FZ:Fe^2+^ complex. Whilst it may initially seem that the average absorbance continues to rise slightly after this point, note that the apparent increase is within error of the measurements taken. From this, it was concluded that the expected absorbance response using these sensing conditions was achieved.

The limit of detection (LoD) and limit of quantification (LoQ) can be determined from the linear section of the graph (Fig. S8†). The LoD and LoQ calculated based on absorbance are 0.18 μM and 0.57 μM, respectively. These values are higher than those quoted in the literature for the ferrozine assay (for example Geißler *et al.* reported an LoD of 1.9 nm for the ferrozine method using microfluidics, and Hopwood *et al.* reported an LoD in the sub-nanomolar region of 0.34 nM using a lab on a chip analyser).^48,51^ However, it is important to note that colorimetric sensors can have a variable ‘working linear range’ depending on the initial sensing conditions chosen by the researcher. The molar extinction coefficient of the 3 : 1 FZ:Fe^2+^ was calculated as 24 390 M^−1^ cm^−1^, which is lower than the literature value of 27 900 M^−1^ cm^−1^ and could possibly account for any minor differences in sensitivity of this probe system. Ultimately, both the LoD and LoQ for the FZ assay determined from the experiments were found to be adequate for further luminescence experiments.

### 3.3 PTIR545 UCNP emission response

The first set of titrations employed the commercially available ultra-bright PTIR545 UCNPs to measure the change in emission response upon addition of Fe^2+^. Similar to the NaYF_4_ UCNPs, the PTIR545 particles were also capped with PAA prior to measurement to maximise particle dispersibility in aqueous solvents. Note that, unlike the NaYF_4_ UCNPs, FTIR analysis did not show the presence of oleic acid in PTIR545 as supplied, and previous analysis has found they are ‘uncapped’, with any weak IR signals assigned to the lattice itself.^52^ Indeed, adopting the same method for ligand substitution outlined in this article still produces PAA-capped particles, as seen in the FTIR spectrum (Fig. S6†), with the peak between 2900–3000 cm^−1^ matching recorded reference data for PAA.

**Fig. 3:**
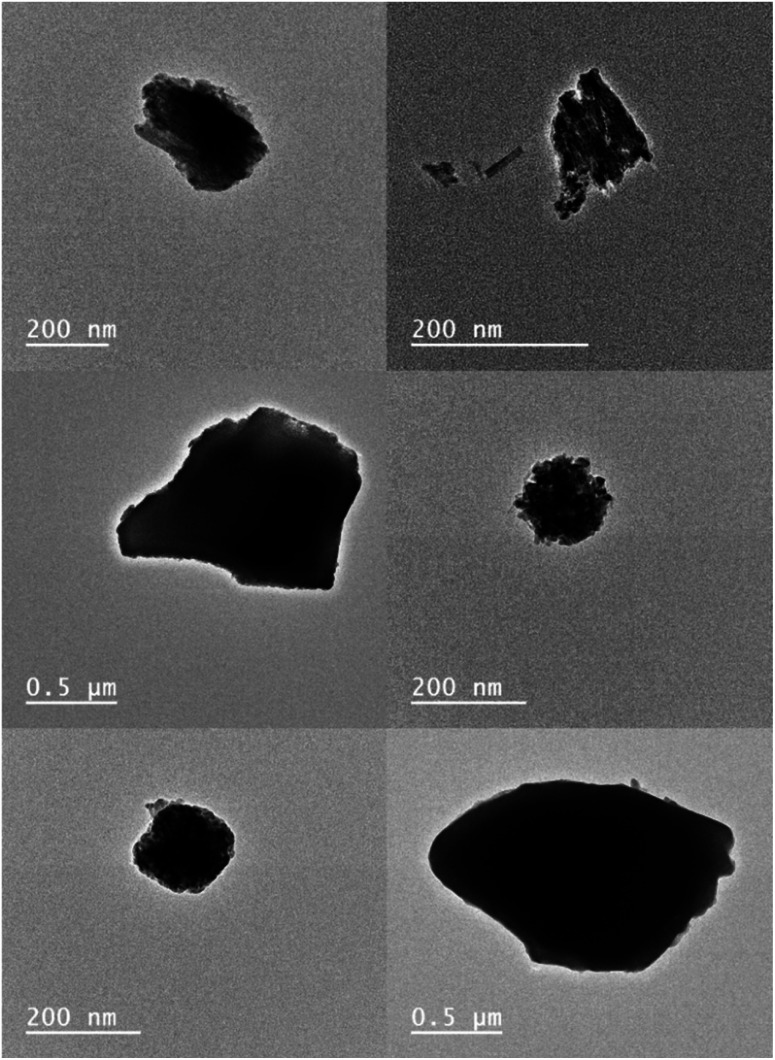
TEM images of PTIR545. Previous analysis by our group (unpublished) has found their average size to be approximately 320 μm by TEM – a 10-fold increase on the typical diameters of NaYF_4_:Yb^3+^,Er^3+^ UCNPs synthesised in our lab. Note that DLS analysis has found the average size to be much larger, at approx. 1.5 μM, but this is with a high degree of uncertainty and the higher value is likely due to aggregation of particles in suspension.^50^

Analysis by TEM (Fig. 3) has shown the size range of PTIR545 to be between 0.2–1 μm, with their large size resulting in a high luminescence intensity simply due to the greater number of luminescent ions present in each particle, when compared to smaller UCNPs.^52,53^ Smaller nanoparticles are typically more desirable for real-world application, but they are inherently less bright compared with larger particles due to the heightened effects of surface quenching on luminescence.^54,55^ Therefore, larger systems such as PTIR545 are ideal for preliminary studies, and we postulated that their high brightness would result in increased sensitivity. However, a major drawback of the commercial PTIR545 UCNPs is their high degree of polydispersity. Typically, the monodispersity of UCNPs decreases any errors between repeat measurements that could be introduced by particle size differences. Therefore, repeat measurements are essential to improve overall reliability.

Particle morphology has also been found to have a marked effect on UCNP luminescence. Spherical morphologies are desirable due to the minimisation of surface area-to-volume ratio, meaning a lower degree of luminescence quenching due to environmental quenchers and surface defects.^53^ While the NaYF_4_:Yb^3+^,Er^3+^ UCNPs shown in Fig. 1 are largely spherical, TEM analysis of PTIR545 shows the overall particle morphology to be somewhat amorphous, with features of both spherical and rod-like morphologies present. However, we believe that, overall, the significantly larger particle size is the major contributing factor to the increased brightness of PTIR545.

**Fig. 4:**
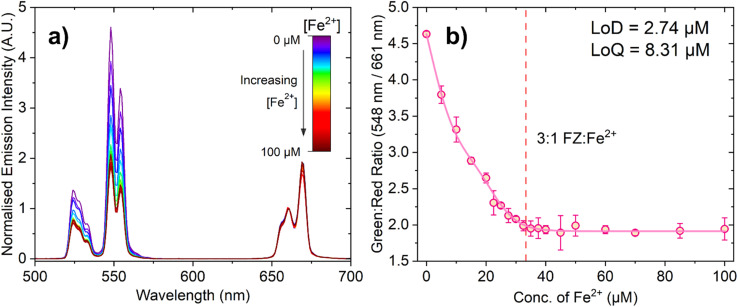
(a) Emission spectrum of ferrozine (100 μM) in pH 5.6 MES buffer (0.1 M) with 150 μL PTIR545 Er^3+^-doped UCNPs with increasing concentration of Fe^2+^ ions. The spectra have been normalised to the peak centred at 661 nm. Similarly to the absorbance measurements, this spectrum shows an example of one repeat, where three repeats were conducted overall. (b) The ratio between the intensity of the green (548 nm) and red (661 nm) peaks at each concentration of Fe^2+^, with the error bars representing standard deviation over three repeats. The red dashed line represents the concentration at which the 3 : 1 FZ:Fe^2+^ complex is expected to form (33.33 μM Fe^2+^). Data fitted using a biphasic dose response function in OriginPro®.

Fig. 4a shows the change in emission intensity of 150 μL of a 10 mg mL^−1^ suspension of PAA-capped PTIR545 UCNPs in the presence of FZ (100 μM) in MES buffer (0.1 M, pH 5.6) with increasing Fe^2+^ concentration. The spectra have been normalised to the peak centred at 661 nm, as this has negligible spectral overlap with the absorbance of the FZ:Fe^2+^ complex, especially when compared to the much stronger overlap of the 548 nm emission band. This demonstrates the ratiometric nature of the probe, allowing for comparison between different nanoparticle batches and negating any *in situ* fluctuations.

Upon low-power laser excitation of the UCNP ferrozine-Fe^2+^ solution at 980 nm, a decrease in emission intensity of the Er^3+^ emission band centred at 548 nm is observed, as the upconverted light emitted by the UCNPs is absorbed by the FZ:Fe^2+^ complex. As the concentration of Fe^2+^ is increased and more FZ:Fe^2+^ complex is formed, the degree of quenching increases in a manner close to linear. Fig. 4b shows ratiometric analysis of the Er^3+^ emission bands centred at 548 nm and 661 nm, where the decrease in the green emission intensity is quantified relative to the red emission band. Once again, the dashed line represents the point at which the solution should be fully saturated with the 3 : 1 FZ:Fe^2+^ complex, and the data is in agreement with the absorption titration data.

The calculated LoD and LoQ values for this probe are 2.74 μM and 8.31 μM, respectively. The observed lower sensitivity of this UCNP-based probe relative to the absorbance-based assay can be attributed to caveats of working with larger UCNPs, such as aggregation and settling out of suspension, and perhaps the very slight spectral overlap of the Er^3+^ 661 nm emission band with the lower energy tail of the 3 : 1 FZ:Fe^2+^ complex absorption band (although we do not expect the latter to be as significant). Nevertheless, despite the fact this UCNP sensing system has a somewhat lower sensitivity than the colorimetric probe based on absorbance, it is still highly sensitive to Fe^2+^, produces a response than can be detected at suitably low concentrations required for Fe^2+^ detection with low laser powers.^48,51^

One major disadvantage of colorimetric sensors based on absorbance is that samples must be optically transparent, as high scatter produces inadequate results. This therefore necessitates that several preparation steps may be necessary before conducting the assay, such as filtration or chemical breakdown of contaminants and pre-concentration.^3^ However, subsequent measurements have provided evidence that the UCNP-based probe is operable in turbid solutions (Fig. S20 and S21†) without any further preparation steps; to the best of our knowledge, this is the first time the FZ assay has been applied to turbid solutions. For these experiments, a suspension of milk powder was added to the cuvette in order to increase turbidity. No indication that the milk powder interacted chemically with the UCNPs, FZ or Fe^2+^ was evident. Repeated measurements using an fs pulsed laser resulted in calculated LoDs of 9.99 μM without milk powder suspension present, and 13.43 μM with milk powder suspension added to form a turbid solution. While there is a clear decrease in sensitivity relative to the experiments in optically transparent solution, these results imply that the probe is able detect micromolar concentrations of Fe^2+^ in turbid solutions. However, it is evident that further optimisation is still required, as these values are higher than the results obtained with optically transparent solutions, as well as the nanomolar LoDs derived from other methods such as those reported by Geißler and Hopwood outlined in Section 3.2.^48,51^

### 3.4 NaYF_4_:Yb^3+^,Er^3+^ emission response

While the increased brightness of PTIR545 was a major advantage for the inner filter measurements, NaYF_4_ UCNPs are the most popular UCNP in the literature due to their ease of synthesis and efficient upconversion. Attention therefore turned to measuring the changing emission response with much smaller PAA-capped NaYF_4_:Yb^3+^,Er^3+^ UCNPs for comparison. We initially theorised that using the smaller UCNPs should mitigate some of the issues introduced by the larger, polydisperse PTIR545 nanoparticles, such as increased aggregation and settling out of suspension, and that this probe may therefore show increased sensitivity to the PTIR545 system. Note that for these experiments, all conditions, concentrations and volumes were identical to the experiments performed with the commercial PTIR545 phosphors, with the only significant difference being the larger emission monochromator slit width used for detection (13 nm, as opposed to 4 nm), which accounts for the lower spectral resolution.

**Fig. 5:**
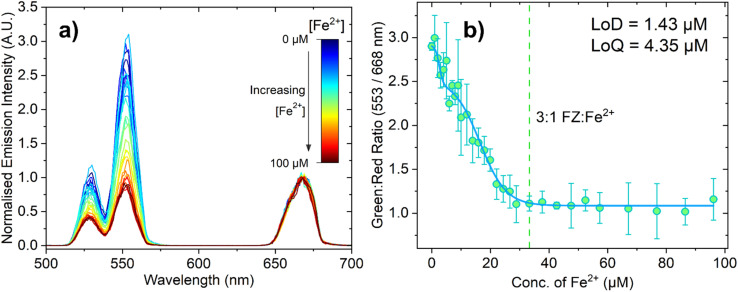
(a) Emission spectrum of ferrozine (100 μM) in pH 5.6 MES buffer (0.1 M) with 150 μL NaYF_4_:Yb^3+^,Er^3+^-doped UCNPs with increasing concentration of Fe^2+^ ions. The spectra have been normalised to the peak centred at 668 nm. This spectrum shows an example of one repeat, where three repeats were conducted overall. The emission monochromator bandwidth was set to 13 nm. (b) The ratio between the intensity of the green (554 nm) and red (668 nm) peaks at each concentration of Fe^2+^, with the error bars representing standard deviation over three repeats. The green dashed line represents the concentration at which the 3 : 1 FZ:Fe^2+^ complex is expected to form (33.33 μM Fe^2+^). Data fitted using a biphasic dose response function in OriginPro®.

Fig. 5a shows the decrease in the normalised emission intensity of the UCNPs (following 980 nm excitation) when the concentration of Fe^2+^ is increased. Analogous to the titrations with the PTIR545 UCNPs, the data was normalised to the red peak, which in this case was centred at 668 nm. We attribute the slight bathochromic shift relative to PTIR545 to slight differences in crystal field effects between the host matrices, as well as lower spectral resolution resulting in individual peaks ‘merging together’.^29^ The high surface area-to-volume ratio of small UCNPs means a greater proportion of their luminescent ions are on (or near) the particle surface. Therefore, their luminescence is more significantly affected by environmental quenching due to solvent and surface ligand vibrations.^28,53^ Higher surface area-to-volume ratios also result in a higher density of surface defects, which have been shown to further lead to non-radiative decay.^56,57^ These factors together necessitate the use of wider spectral bandwidths to adequately detect the emission, resulting in lower spectral resolution. Fig. 5b shows the ratiometric analysis of the green and red peaks and, again, the dashed line represents the point at which the 3 : 1 FZ:Fe^2+^ complex is expected to be dominant. The response appears to plateau after this point, suggesting the solution is fully saturated with the 3 : 1 FZ:Fe^2+^ complex, which is in agreement with both the absorbance and PTIR545 titration data. It is apparent from Fig. 5b that these experiments suffer from much larger errors than the PTIR545 experiments, which we attribute to increased scatter due to the wider spectral bandwidth employed. The LoD and LoQ for this probe were calculated as 1.43 μM and 4.35 μM, respectively. This higher sensitivity achieved using the synthesised nanometre-sized NaYF_4_ particles agrees with the hypothesis that smaller particles with higher dispersibility may result in increased sensitivity. These results are noteworthy and show that a sensitive Fe^2+^ probe based on upconverted luminescence is possible to achieve based not only on ultra-bright particles, but also on small, easily synthesised nanoparticles.

Analogously to the PTIR545 UCNPs, we also conducted preliminary turbid solution measurements in the presence of milk using an fs pulsed laser (Fig. S22 and S23†) under 980 nm excitation. Much like the Fe^2+^ quantification experiments using a low-power CW laser, the smaller NaYF_4_ UCNPs displayed a higher sensitivity under these conditions when compared to PTIR545, with LoDs calculated as 7.47 μM without the presence of milk powder suspension and 5.39 μM in turbid solution. In contrast to the PTIR545 measurements, the LoD in turbid solution is lower (Fig. S23a†). Again, further optimisation is necessary to improve the sensitivity of this system and allow advanced, accurate turbid solution Fe^2+^ detection using low-power lasers.

### 3.5 Competing ions

**Fig. 6:**
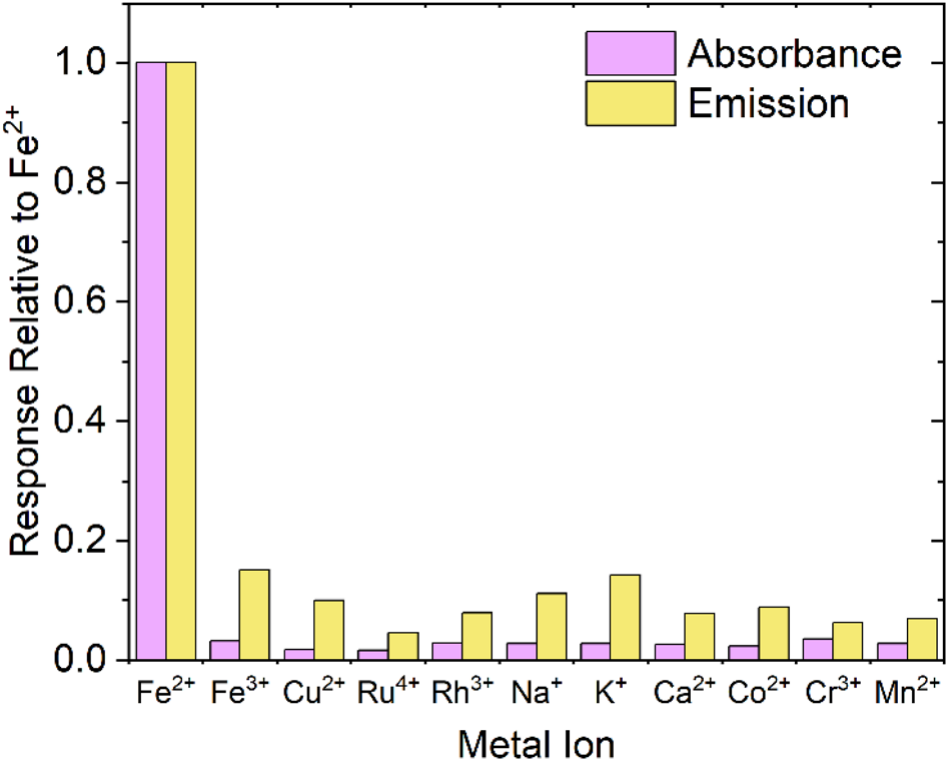
The response of other common metal ions (Fe^3+^, Cu^2+^, Ru^4+^, Rh^3+^, Na^+^, K^+^, Ca^2+^, Co^2+^, Cr^3+^, Mn^2+^), with the ferrozine assay (absorbance) and ferrozine-UCNP assay (emission, using PAA-capped PTIR545 UCNPs), relative to the response produced by Fe^2+^.

When designing a probe for a specific analyte, it is obviously important to test that the response is specific to the desired analyte. Fig. 6 shows both the absorbance and emission response of FZ to several common ions present in groundwater/biological media, and competing or potentially optically interfering ions (Fe^3+^, Cu^2+^, Ru^4+^, Rh^3+^, Na^+^, K^+^, Ca^2+^, Co^2+^, Cr^3+^, Mn^2+^), up to an excess of 150 μM (>5 equivalents), relative to the response displayed by addition of Fe^2+^. For emission measurements, PTIR545 UCNPs were chosen due to their superior brightness, but we postulate comparable results would be achieved using NaYF_4_:Yb^3+^,Er^3+^ UCNPs. From Fig. 6, it is clear the response produced by addition of Fe^2+^ is far more distinct than the response produced by any other potential competing analytes and, overall, the probe is over 5 times more sensitive to Fe^2+^ than other competing analytes.

For each competing cation, the change in absorbance is clearly negligible relative to the response produced by Fe^2+^, but the change in emission appears to be more significant. This is attributed to the aforementioned errors introduced by the larger UCNPs including aggregation, settling out of solution and size differences due to high polydispersity. Note that there is no detectable colour change with any analyte other than Fe^2+^, leading us to conclude that the probe is highly selective for Fe^2+^.

### 3.6 Improving sensitivity

Whilst we are satisfied that these results demonstrate the detection of sufficiently low concentrations of Fe^2+^ using low-power lasers, ongoing work is focussing on synthesising brighter core@shell NaYF_4_:Yb^3+^,Er^3+^ UCNPs. These nanoparticle architectures have been shown to increase brightness by passivating the particle surface with an inert NaYF_4_ shell, preventing non-radiative deactivation of the luminescence by solvent or ligand vibrations.^58,59^ Other methods involve doping Yb^3+^ ions into the shell to further enhance particle brightness, and introducing Nd^3+^ dopants to shift the excitation wavelength to 808 nm in order to decrease attenuation of excitation light in aqueous media.^60,61^ It is therefore conceivable that this increased brightness, combined with the stability in suspension of smaller PAA-capped particles, will further improve the sensitivity of this Fe^2+^ probe and other related optical detection systems in the future.

It is worth noting that in some cases, the visible absorbance of the FZ:Fe^2+^ complex can be considered a drawback that results in a loss of sensitivity, as in ‘real’ samples other components with absorption in the visible region may interfere with results, particularly in biological media. Whilst we did not find this necessary for our preliminary studies, we propose that moving towards a RET-based system by covalently attaching the Fe^2+^ ion selective colorimetric chelate will obviate this and allow the use of lifetime analysis to investigate binding.^6^ Noting that covalent attachment of acceptor species to UCNPs is often difficult due to the requirement to homogeneously cap the particles with a silica shell, we aimed to capitalise on simplicity and ease of implementation.^25^ The colorimetric ferrozine assay has been ubiquitous throughout the literature for decades despite its drawbacks, and still displays both high sensitivity and selectivity regardless.

## 4. Conclusions

We have successfully demonstrated the use of the commercially readily available reagent FZ in conjunction with UCNPs to design metal ion probes for Fe^2+^ ions, based on inner filter effects arising from overlap of the FZ:Fe^2+^ complex absorbance with emission from Er^3+^-doped UCNPs. The absorbance of a solution of FZ in buffer increases as Fe^2+^ concentration is increased, and when UCNPs are added the green emission is quenched ratiometrically due to the IFE. The probes display fast response times and high sensitivity (displayed by the LoDs of 2.74 μM and 1.43 μM for the PTIR545 and NaYF_4_:Yb^3+^,Er^3+^ UCNPs, respectively) and shows minimal response with other competing ions. While the commercially available PTIR545 nanoparticles display well-resolved spectra due to their large size and improved brightness, the synthesised NaYF_4_:Yb^3+^,Er^3+^ UCNPs display comparable results and have the benefit of being easily synthesisable in any chemistry lab. It is expected that, due to the low cost and high availability of FZ, this sensor could have potential application in Fe^2+^ sensing in settings including biological tissue; work towards this is currently in progress, including development of RET-based detection systems.

## Author contributions

RA, LN and HW designed the experiments, MN provided hands-on training on nanoparticle synthesis, RA performed all the experiments and analysed the data, and RA and LN wrote the manuscript.

## Conflicts of interest

There are no conflicts to declare.

## Supplementary Material

RA-013-D3RA04645A-s001
