# Supplementary material for: Development of an Fe2+ sensing system based on the inner filter effect between upconverting nanoparticles and ferrozine
Source: RSC Adv. 2023 Sep 4;13(37):26313–22. doi: 10.1039/d3ra04645a (PMC10475975; doi:10.1039/d3ra04645a)
Supplement: RA-013-D3RA04645A-s001 [file RA-013-D3RA04645A-s001.pdf]

Electronic Supplementary Material (ESI) for RSC Advances.
This journal is © The Royal Society of Chemistry 2023

Development of an Fe^{2+} Sensing System Based on the Inner Filter Effect Between Upconverting Nanoparticles and Ferrozine

Ruth Abramson, Hannah Wilson, Marta M. Natile and Louise S. Natrajan

Supporting Information

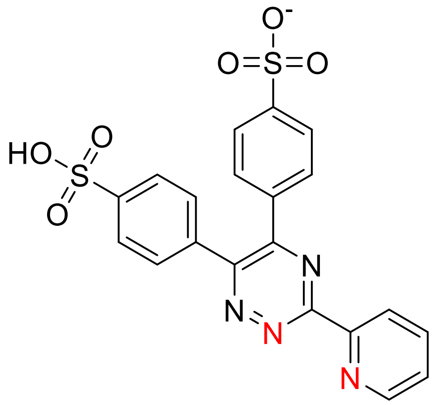

Figure S1: The structure of ferrozine. Ligand binding to Fe^{2+} occurs *via* the nitrogen atoms coloured in red, in a 3:1 ratio.

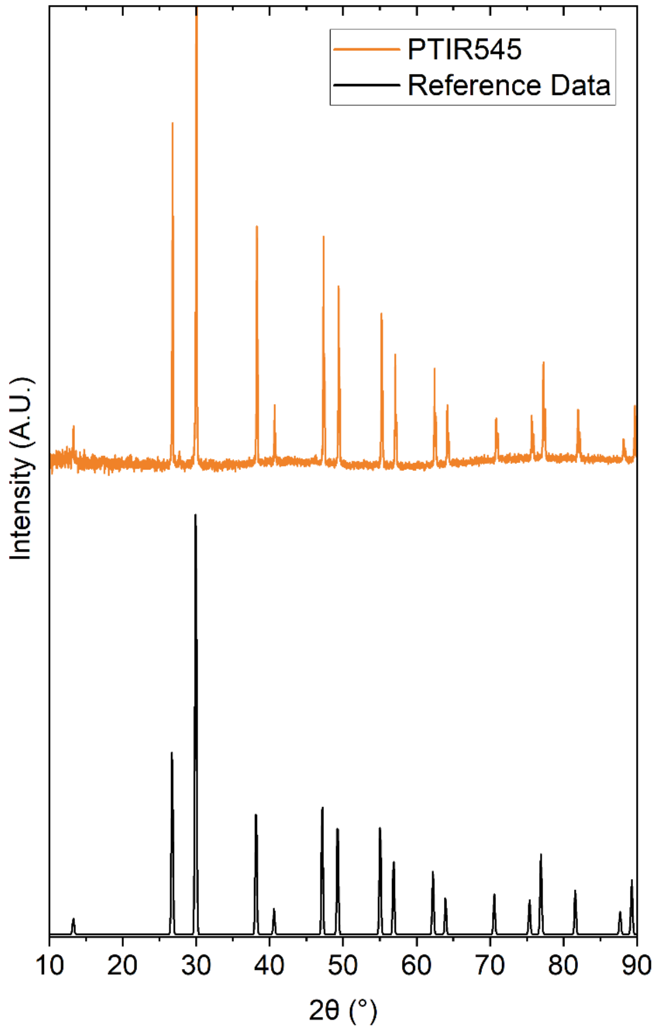

Figure S2: Powder X-ray diffraction data for PTIR545 compared to reference data from the International Crystal Structure Database (ICSD No. 230767) for Gd_2S_2O. Full methodology for pXRD data collection can be found in the main text. Data has been baseline corrected using the OriginPro® software.

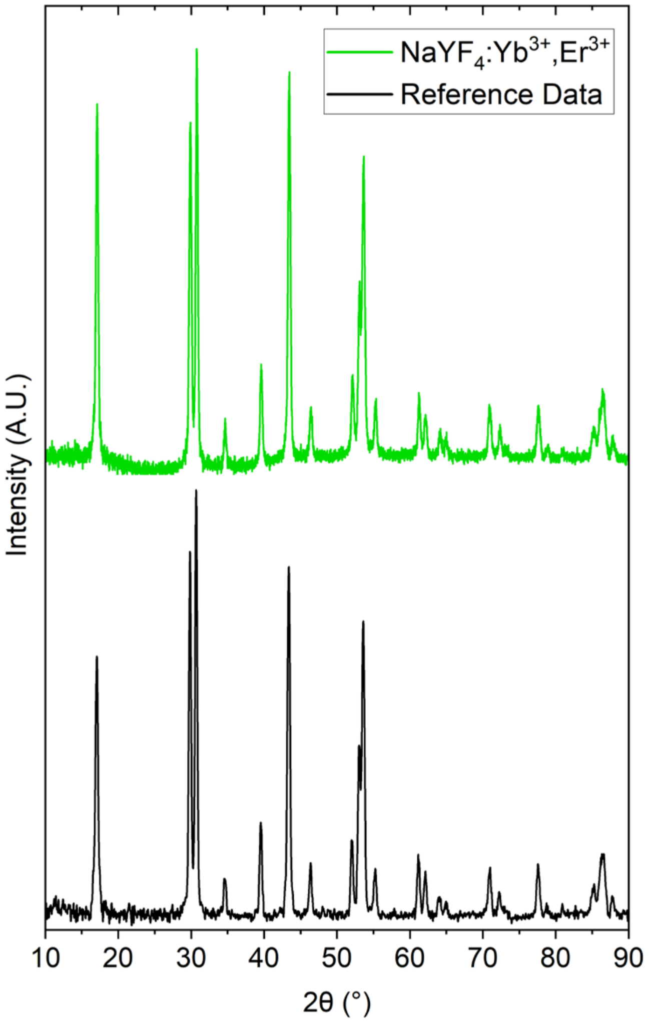

Figure S3: Powder X-ray diffraction pattern data of $NaYF_4:Yb^{3+},Er^{3+}$ UCNPs synthesised in our lab compared to reference data from the International Crystal Structure Database (ICSD no. 51916) for β-phase $NaYF_4$. Full methodology for pXRD data collection can be found in the main text. Data has been baseline corrected using the OriginPro® software.

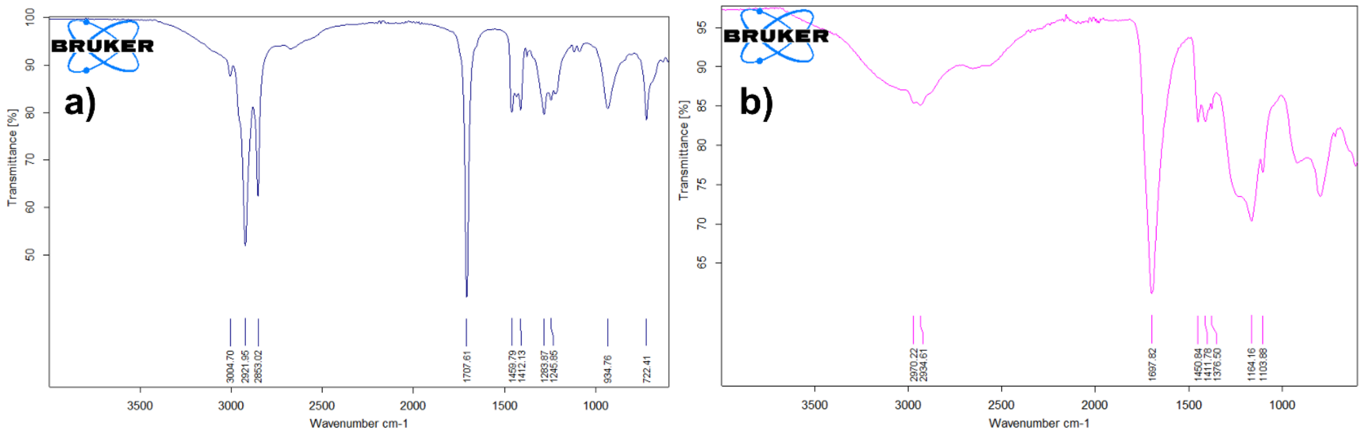

Figure S4: FTIR spectra of a) oleic acid and b) PAA as purchased. The strong carbonyl stretch at approx. 1700 cm^{-1} is visible in both cases.

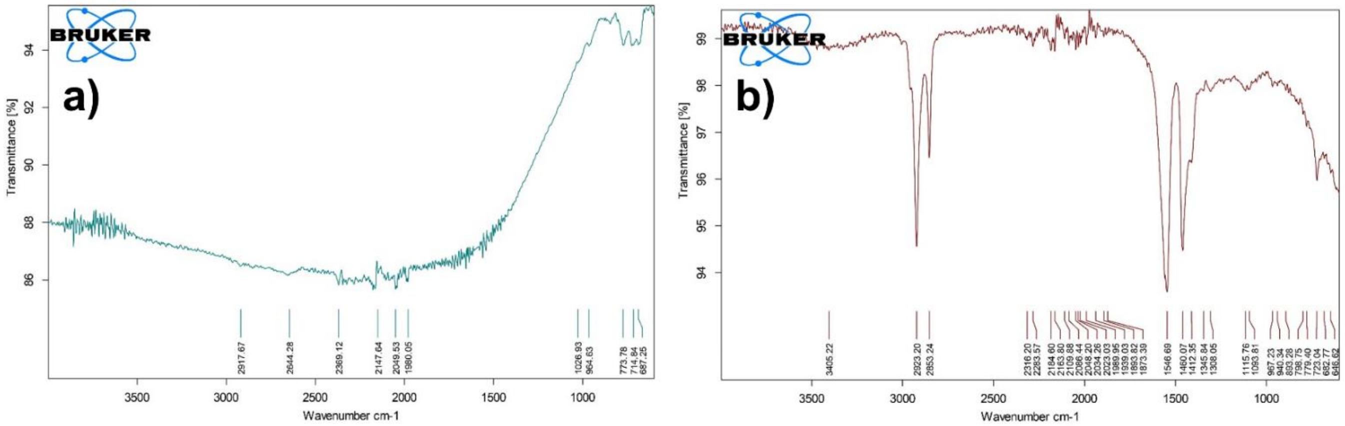

Figure S5: a) FTIR spectrum of PTIR545 UCNPs (unmodified), measured as a solid powder. Any small peaks are assigned to the ionic lattice. b) FTIR spectrum of OA-capped $NaYF_4$:Yb^{3+},Er^{3+} UCNPs (UCNPs as synthesised), measured as a solid powder. Note the disappearance of the carbonyl stretch at 1708 cm^{-1}, indicating oleic acid is bound to the UCNP surface via the carboxylate group.

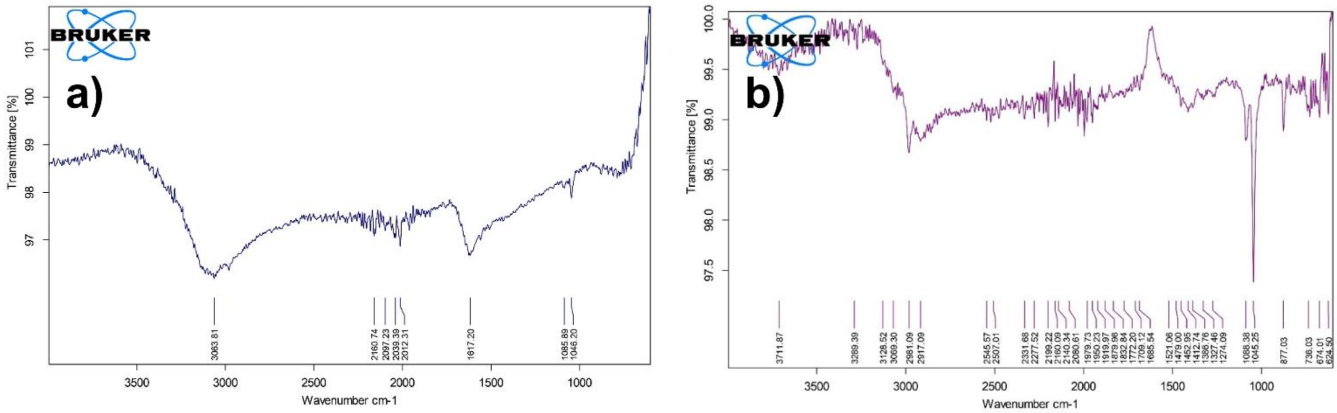

Figure S6: FTIR spectra of suspensions of a) PAA-capped PTIR545 and b) PAA-capped $NaYF_4$:Yb^{3+},Er^{3+} UCNPs. Water has been subtracted from the background in both cases. In both cases, we attribute the broad peak centred at approx. 3000 cm^{-1} to the presence of PAA.[42]

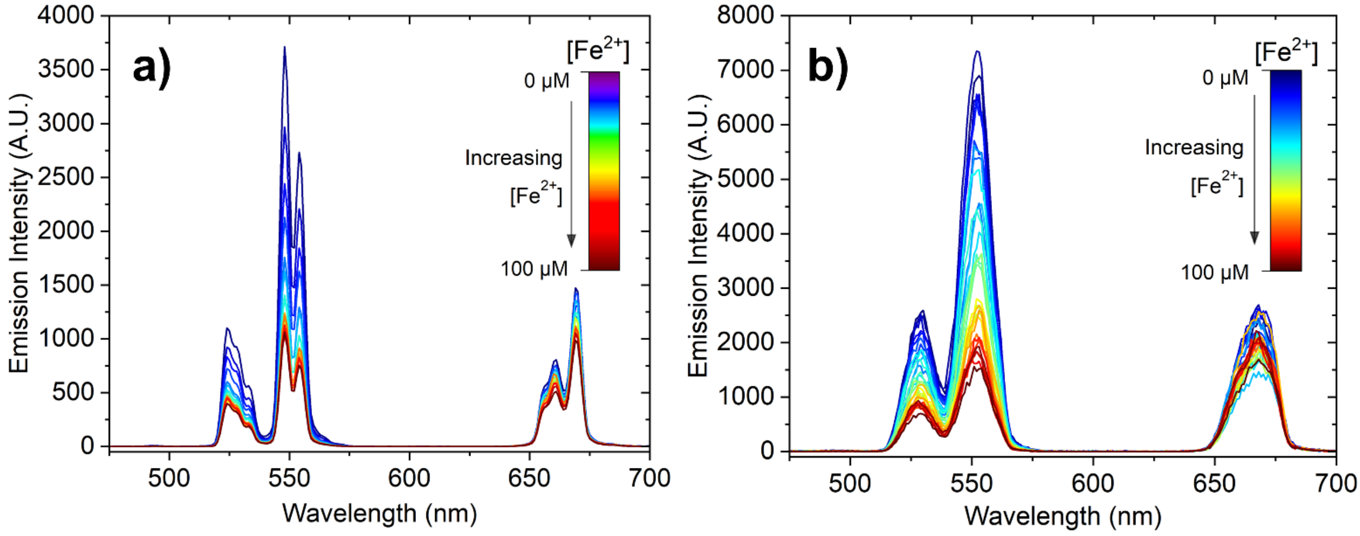

Figure S7: Examples of emission spectra recorded from the Fe^{2+} titrations with a) PTIR545 and b) $NaYF_4$:Yb^{3+},Er^{3+}. The spectra are shown as recorded (prior to normalisation).

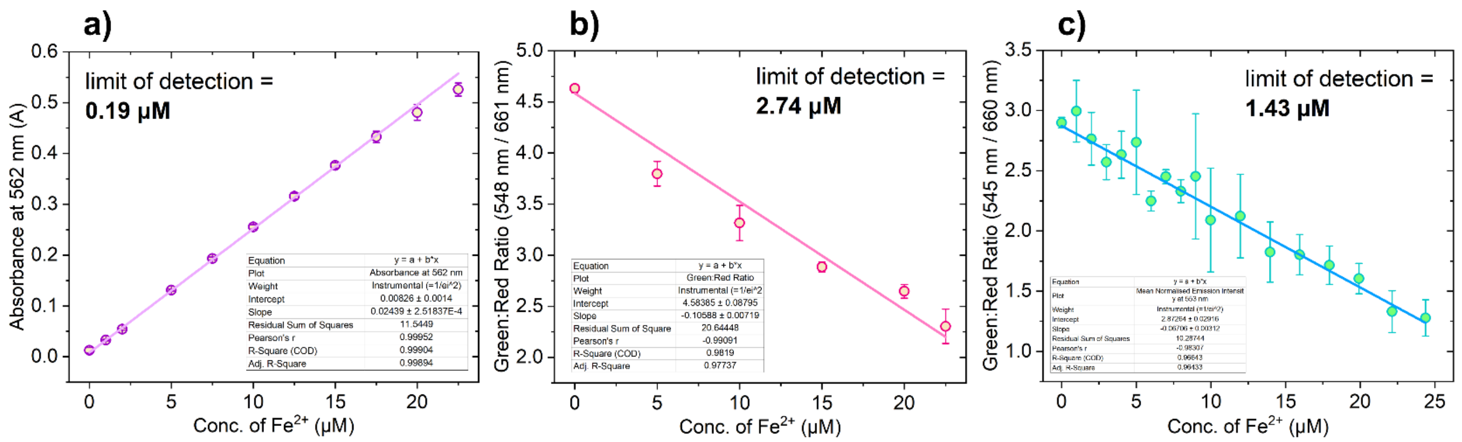

Figure S8: The linear sections of the plots used to calculate the LoD/LoQ from a) the absorbance measurements; b) the PTIR545 measurements and c) the $NaYF_4$:Yb^{3+},Er^{3+} measurements. Values were calculated based on the error of the intercept as given by the regression fits.

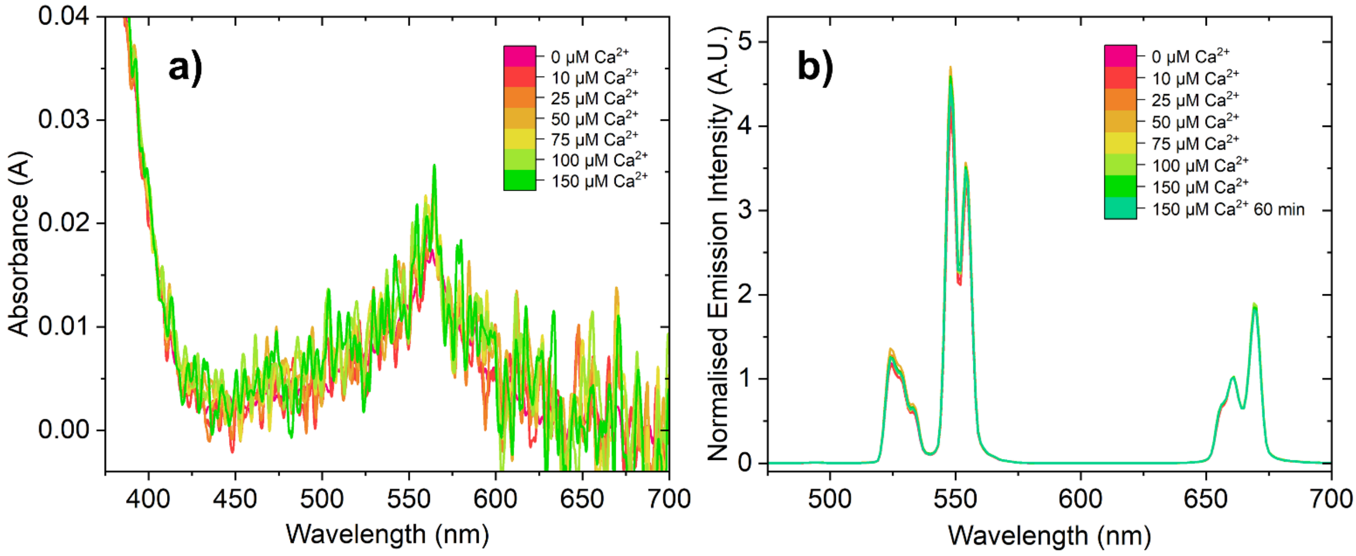

Figure S9: The absorbance (a) and emission (b) spectra for the titrations carried out between FZ, PTIR545 UCNPs (emission only) and Ca^{2+} ions to determine the degree of competing response.

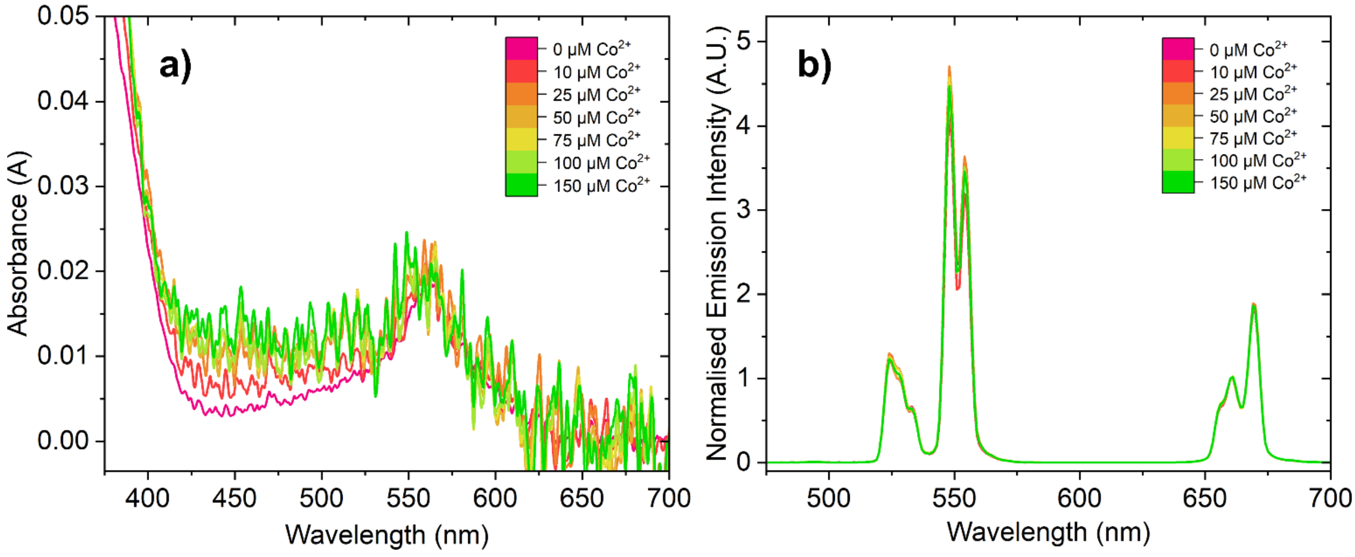

Figure S10: The absorbance (a) and emission (b) spectra for the titrations carried out between FZ, PTIR545 UCNPs (emission only) and Co^{2+} ions to determine the degree of competing response.

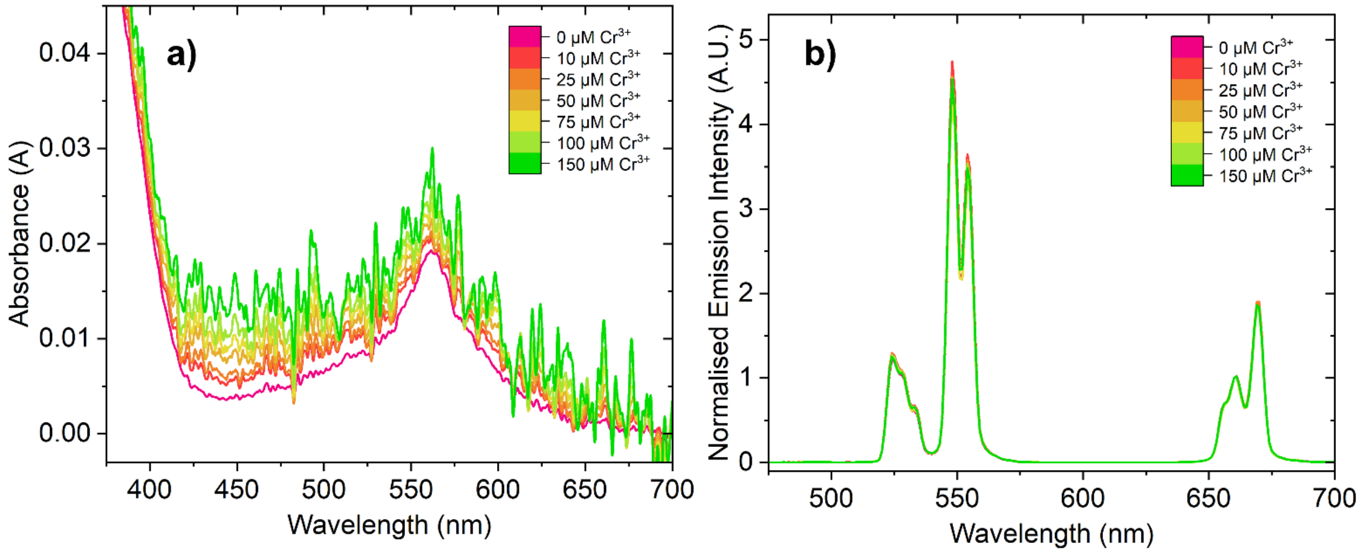

Figure S11: The absorbance (a) and emission (b) spectra for the titrations carried out between FZ, PTIR545 UCNPs (emission only) and Cr^{2+} ions to determine the degree of competing response.

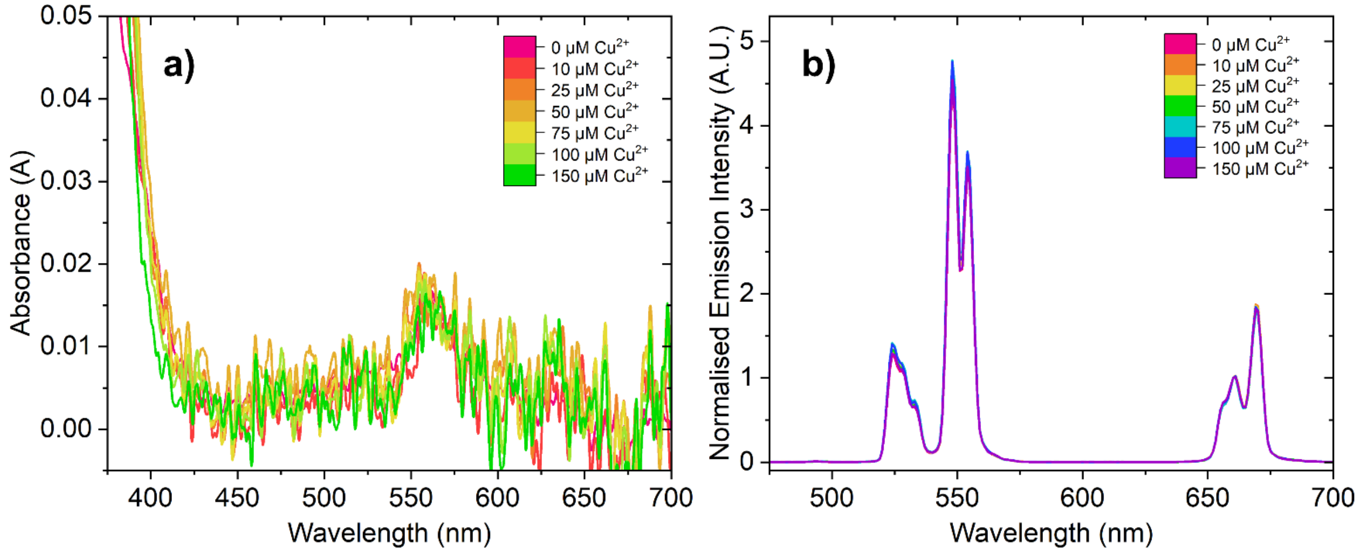

Figure S12: The absorbance (a) and emission (b) spectra for the titrations carried out between FZ, PTIR545 UCNPs (emission only) and Cu^{2+} ions to determine the degree of competing response.

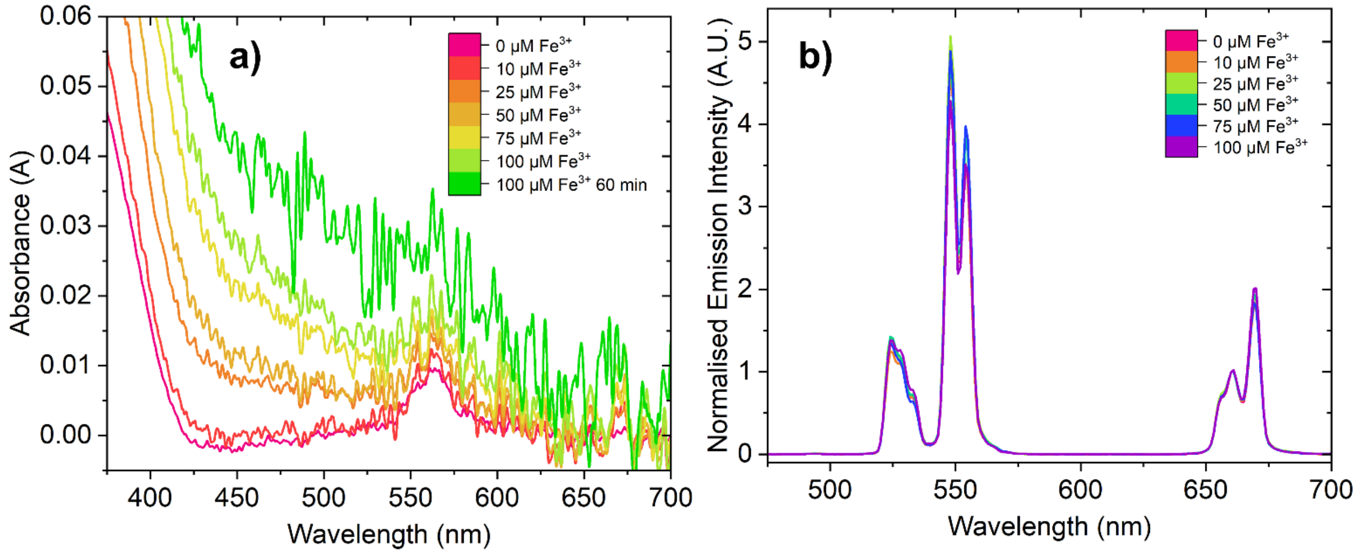

Figure S13: The absorbance (a) and emission (b) spectra for the titrations carried out between FZ, PTIR545 UCNPs (emission only) and Fe^{3+} ions to determine the degree of competing response.

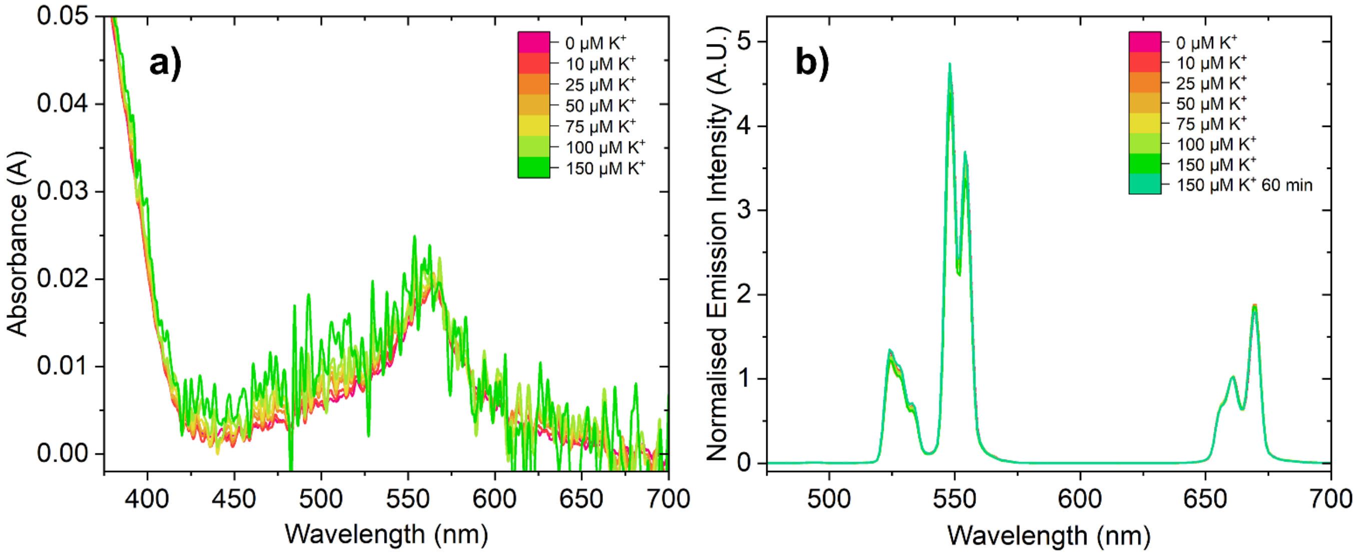

Figure S14: The absorbance (a) and emission (b) spectra for the titrations carried out between FZ, PTIR545 UCNPs (emission only) and K^+ ions to determine the degree of competing response.

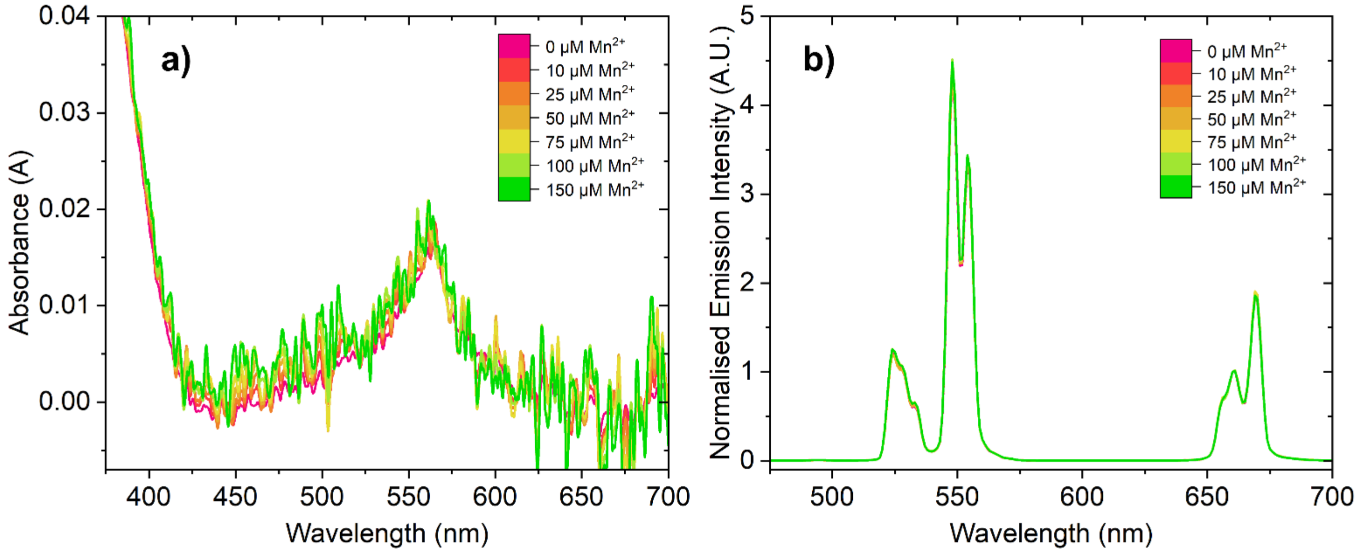

Figure S15: The absorbance (a) and emission (b) spectra for the titrations carried out between FZ, PTIR545 UCNPs (emission only) and Mn^{2+} ions to determine the degree of competing response.

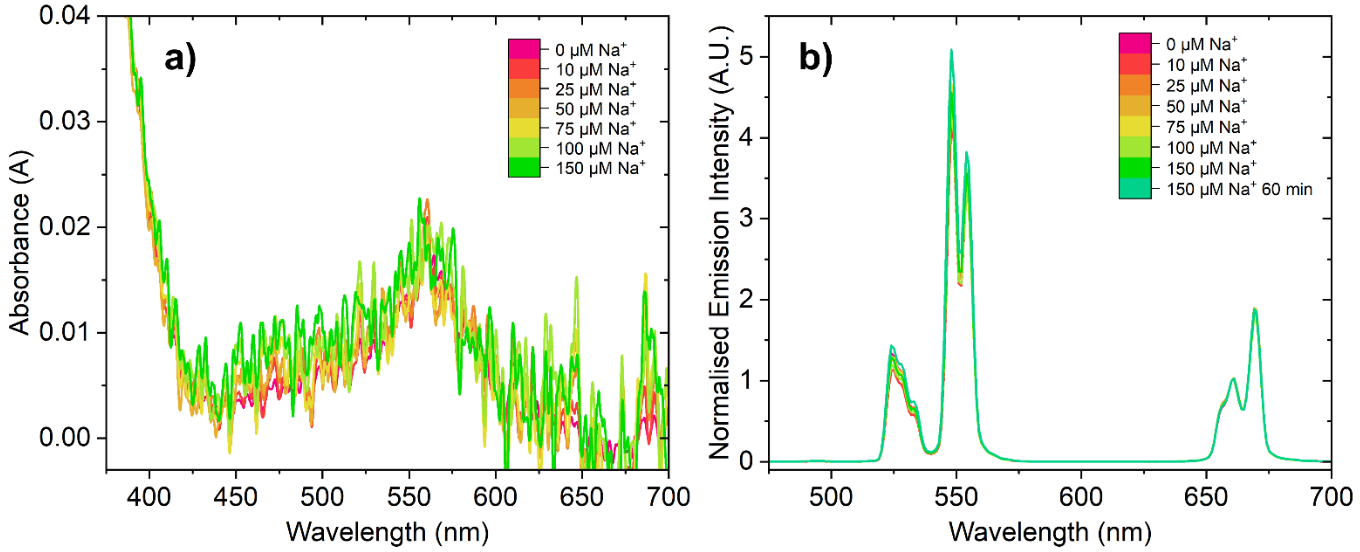

Figure S16: The absorbance (a) and emission (b) spectra for the titrations carried out between FZ, PTIR545 UCNPs (emission only) and Na^+ ions to determine the degree of competing response.

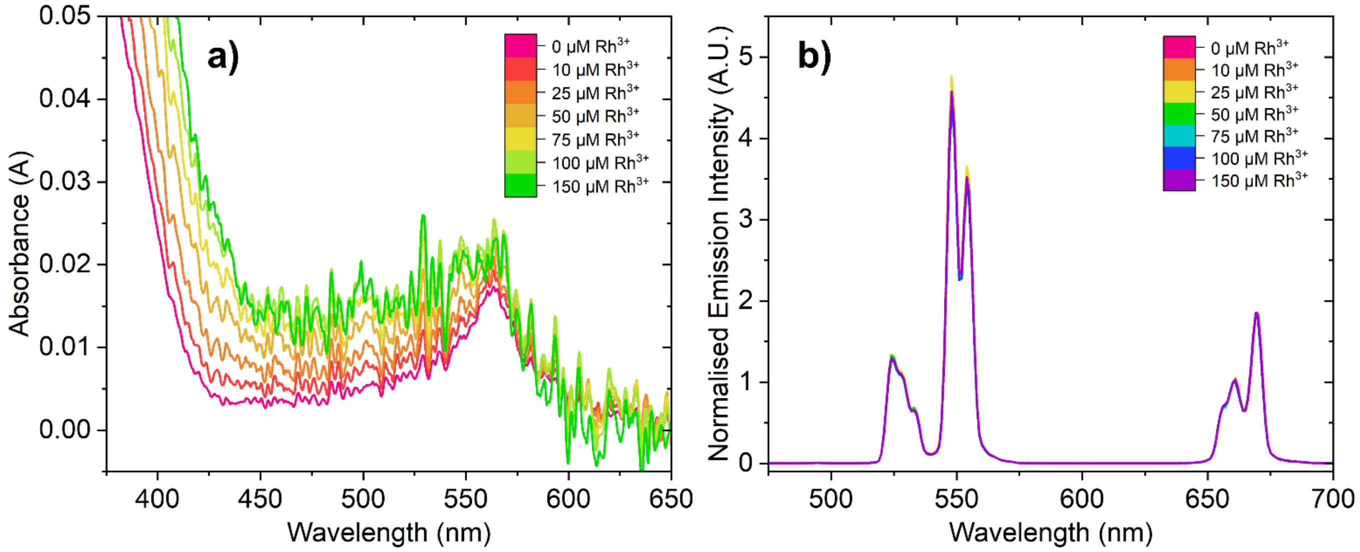

Figure S17: The absorbance (a) and emission (b) spectra for the titrations carried out between FZ, PTIR545 UCNPs (emission only) and Rh^{3+} ions to determine the degree of competing response.

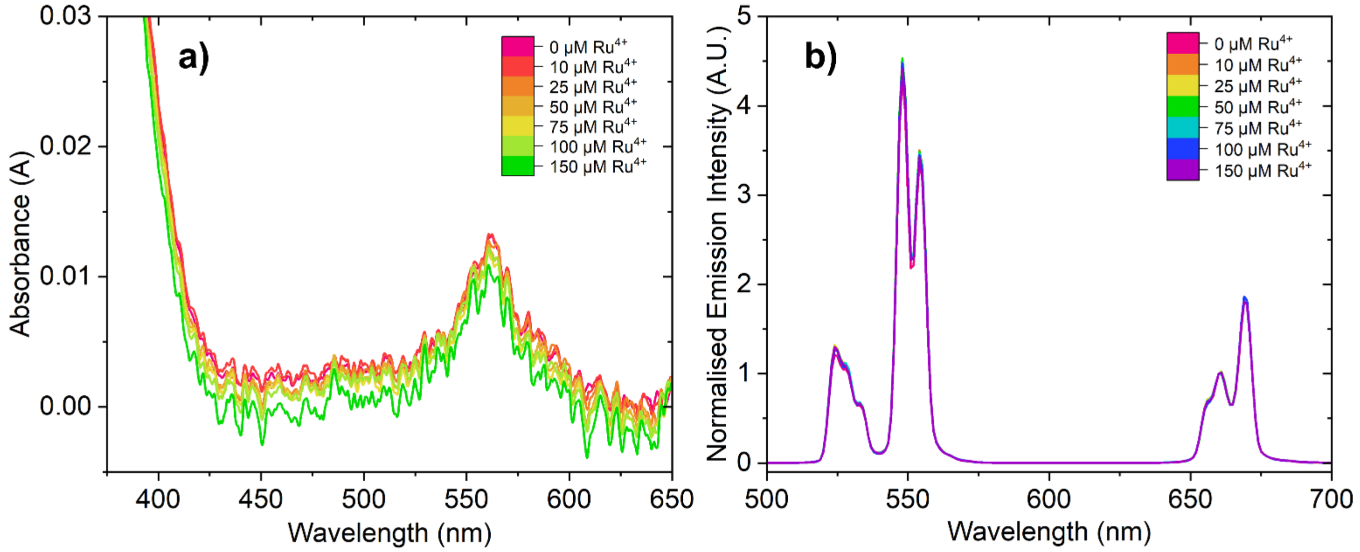

Figure S18: The absorbance (a) and emission (b) spectra for the titrations carried out between FZ, PTIR545 UCNPs (emission only) and Ru^{4+} ions to determine the degree of competing response.

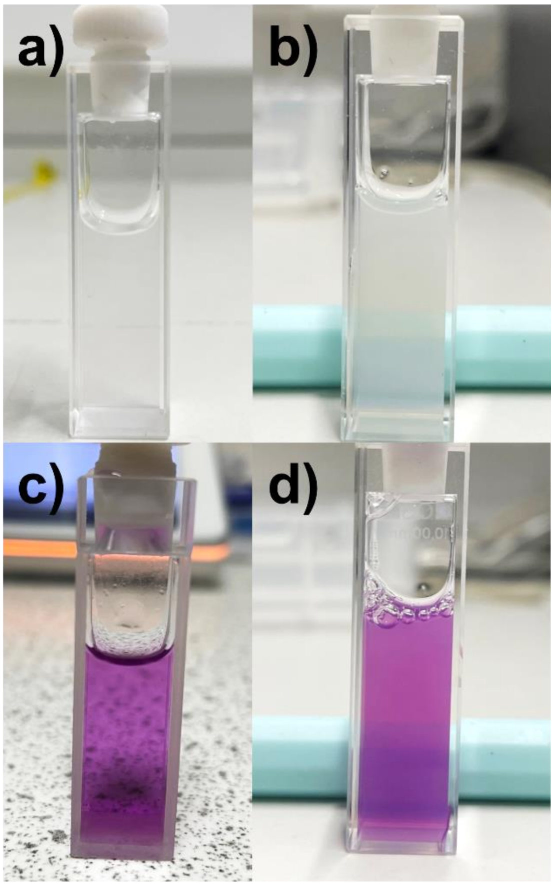

Figure S19: Images of the cuvettes used for the turbid solution measurements at both the start (a and b) and end (c and d) points of the titrations. a and c contain no milk powder suspension; b and d contain 50 µL milk.

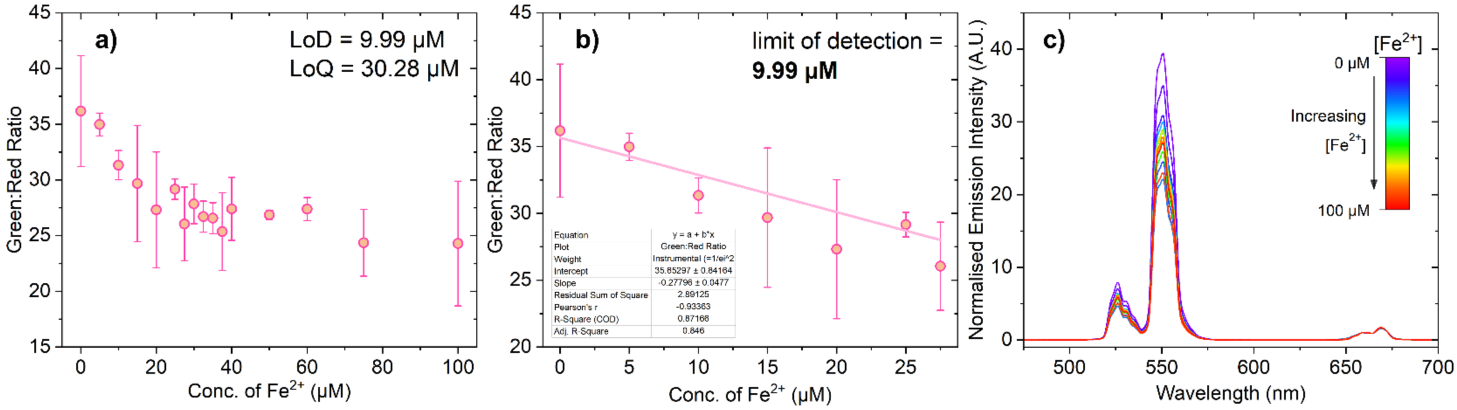

Figure S20: a) The green-to-red ratio between the emission peaks of PTIR545 from titrations in the presence of FZ (100 µM) with increasing [Fe^{2+}]. Error bars represent standard deviation over 3 repeats; b) the linear section of the graph used to calculate the limit of detection and c) the normalised emission spectrum. Note this is an example of spectra obtained from 1 titration; three repeat titrations were conducted. These measurements were recorded using an ultra-fast fs pulsed laser; we theorise that fluctuations in power density contribute to the large errors, as well as errors with aggregation/settling as discussed in the main text, resulting in a lower LoD/Q — we believe this is also the case for all other measurements recorded using the fs pulsed laser shown in Figures S21-23.

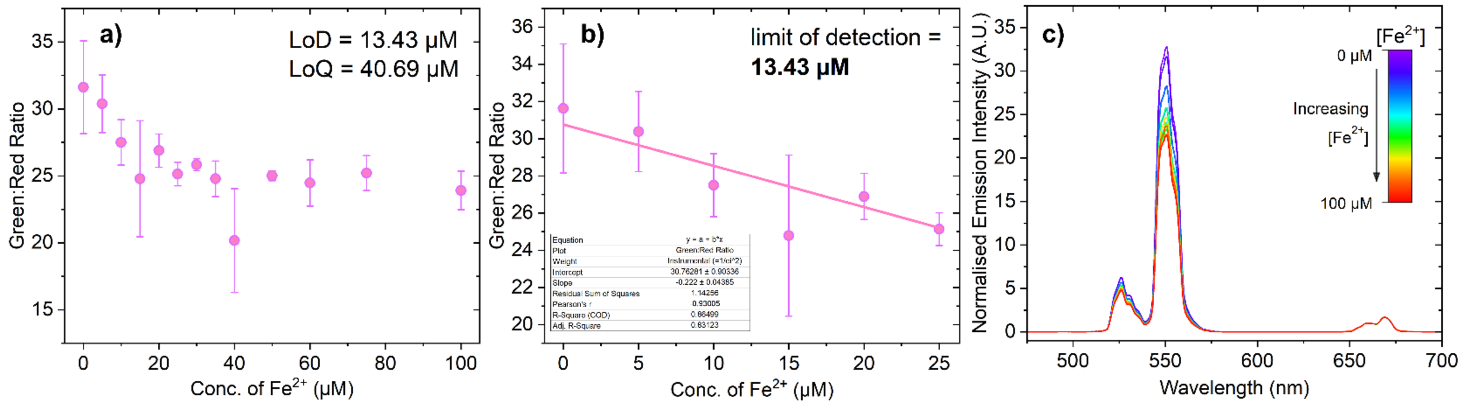

Figure S21: a) The green-to-red ratio between the emission peaks of PTIR545 from titrations in the presence of FZ (100 µM) and 50 µL milk powder suspension with increasing [Fe^{2+}]. Error bars represent standard deviation over 3 repeats; b) the linear section of the graph used to calculate the limit of detection and c) the normalised emission spectrum. Note this is an example of spectra obtained from 1 titration; three repeat titrations were conducted. These measurements were recorded using an ultra-fast fs pulsed laser; again, we theorise that fluctuations in power density contribute to the large errors, as well as errors with aggregation/settling as discussed in the main text, resulting in a lower LoD/Q.

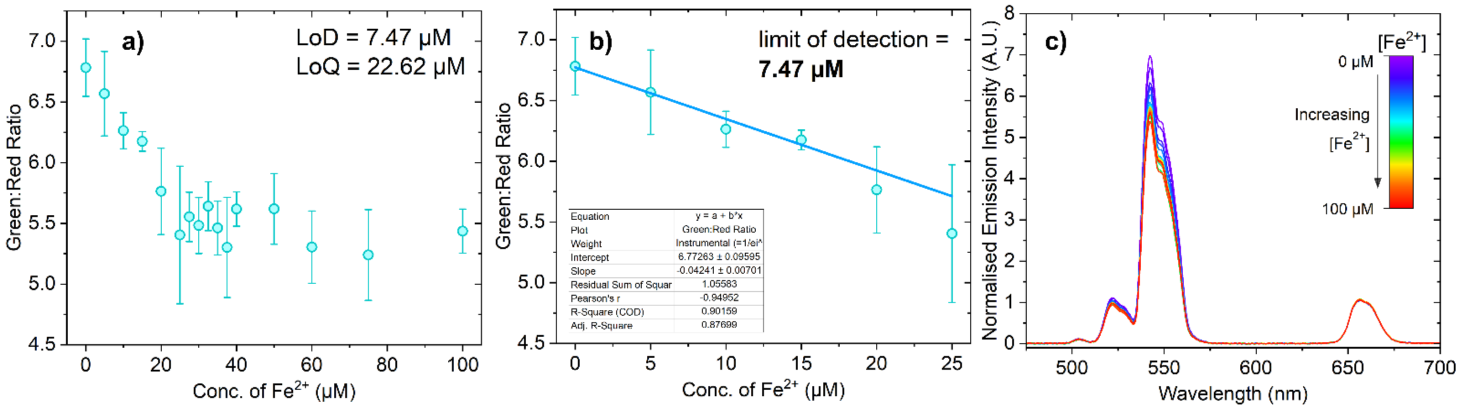

Figure S22: a) The green-to-red ratio between the emission peaks of $NaYF_4$:Yb^{3+},Er^{3+} from titrations in the presence of FZ (100 µM) with increasing [Fe^{2+}]. Error bars represent standard deviation over 3 repeats; b) the linear section of the graph used to calculate the limit of detection and c) the normalised emission spectrum. Note this is an example of spectra obtained from 1 titration; three repeat titrations were conducted.

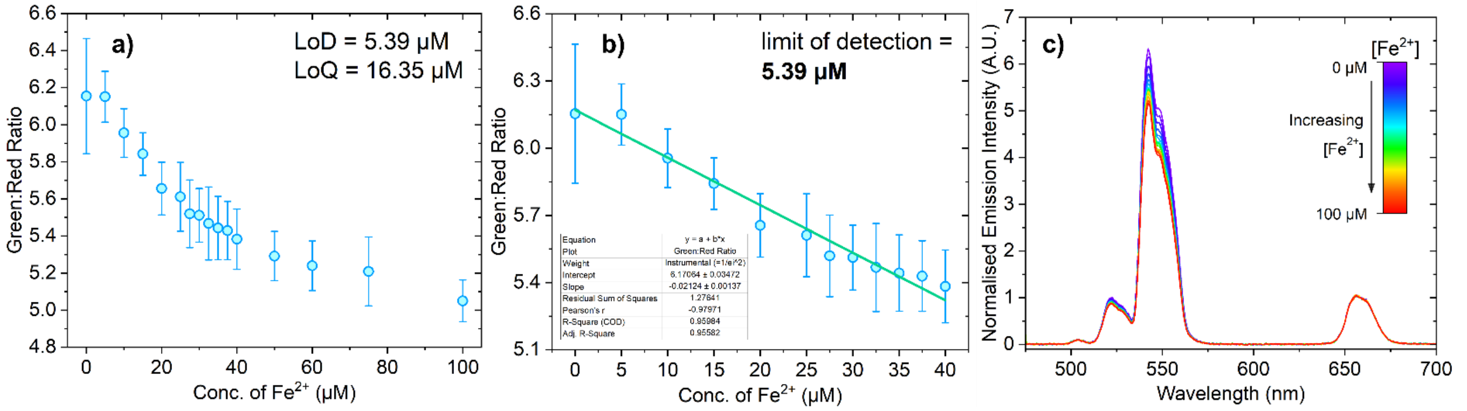

Figure S23: a) The green-to-red ratio between the emission peaks of $NaYF_4$:Yb^{3+},Er^{3+} from titrations in the presence of FZ (100 µM) and 50 µL milk powder suspension with increasing [Fe^{2+}]. Error bars represent standard deviation over 3 repeats. Unlike other green-to-red ratio plots shown throughout this manuscript and supporting information, this plot does not appear to plateau – we attribute this to the presence of milk powder suspension slowing the kinetics of the FZ/Fe^{2+} complexation reaction; b) the linear section of the graph used to calculate the

limit of detection and c) the normalised emission spectrum. Note this is an example of spectra obtained from 1 titration; three repeat titrations were conducted.
